# Astragaloside IV improves slow transit constipation by regulating gut microbiota and enterochromaffin cells

**DOI:** 10.3389/fphar.2023.1196210

**Published:** 2023-11-21

**Authors:** Xingyang Wan, Qian Zhou, Huaxian Chen, Zhen Li, Mianling Mo, Zhimin Liu, Heng Zhang, Zhuojie He, Guozhong Xiao, Yihui Zheng, Hongcheng Lin, Donglin Ren

**Affiliations:** ^1^ Department of General Surgery (Department of Coloproctology), The Sixth Affiliated Hospital, Sun Yat-sen University, Guangzhou, China; ^2^ Guangdong Provincial Key Laboratory of Colorectal and Pelvic Floor Diseases, The Sixth Affiliated Hospital, Sun Yat-sen University, Guangzhou, China; ^3^ Biomedical Innovation Center, The Sixth Affiliated Hospital, Sun Yat-sen University, Guangzhou, China; ^4^ Department of Pathology, First Hospital of Changsha, Changsha, Hunan, China

**Keywords:** astragaloside IV, enterochromaffin cells, Piezo2, 3-BrY, gut microbiota, slow transit constipation

## Abstract

**Purpose:** Slow transit constipation (STC) is a common gastrointestinal disorder characterized by altered gut microbiota and reduced number of enterochromaffin cells (ECs). Astragaloside IV (AS-IV), a low drug permeability saponin, has showed beneficial effects on patients with STC. However, the specific mechanism by which AS-IV regulates STC remains unclear. In this study, we aimed to investigate the effect of AS-IV on STC and its associated mechanisms involving gut microbiota.

**Methods:** The effect of AS-IV on STC was evaluated on STC mice induced with loperamide. We measured defecation frequency, intestinal mobility, ECs loss, and colonic lesions in STC mice treated with AS-IV. We also analyzed the changes in gut microbiota and metabolites after AS-IV treatment. Moreover, we investigated the relationship between specific gut microbes and altered fecal metabolites, such as 3-bromotyrosine (3-BrY). We also conducted *in vitro* experiments to investigate the effect of 3-BrY on caspase-dependent apoptosis of ECs and the activation of the p38 MAPK and ERK signaling pathways induced by loperamide.

**Results:** AS-IV treatment promoted defecation, improved intestinal mobility, suppressed ECs loss, and alleviated colonic lesions in STC mice. AS-IV treatment also affected gut microbiota and metabolites, with a significant correlation between specific gut microbes and altered fecal metabolites such as 3-BrY. Furthermore, 3-BrY may potentially reduce caspase-dependent apoptosis of ECs and protect cell survival by inhibiting the activation of the p38 MAPK and ERK signaling pathways induced by loperamide.

**Conclusion:** Our findings suggest that changes in gut microbiota and ECs mediated the therapeutic effect of STC by AS-IV. These results provide a basis for the use of AS-IV as a prebiotic agent for treating STC. The specific mechanism by which AS-IV regulates gut microbiota and ECs warrants further investigation.

## Introduction

Slow transit constipation (STC) is a prevalent form of functional constipation resulting from colonic transmission dysfunction, reduced bowel movement, and dry stool. Its prevalence among chronic constipation subtypes ranges from 15% to 30% (Pannemans et al., 2020). Due to its intricate symptoms and high recurrence rate, STC treatment is difficult and conventional therapies often yield unsatisfactory results (Bharucha and Lacy, 2020). Thus, elucidating the pathogenesis of STC and identifying more effective treatments are critical research objectives.

Recent research has highlighted the critical role of gut microorganisms in the pathogenesis of STC(Zhang et al., 2021), as the diversity of gut microbiota and their metabolites are significantly linked to colon transport function. Traditional Chinese medicine has been found to improve defecation by enhancing gut microbiota composition, remodeling related metabolites, increasing intestinal mucin secretion, and promoting intestinal peristalsis (Xu et al., 2019). Studies have shown that astragalus decoction promotes beneficial bacterial colonization and improves intestinal peristalsis in rats, and the main active compound, astragaloside IV (AS-IV), enhances intestinal movement (Liu et al., 2022). Our previous study on AS-IV treated STC mice demonstrated microbiota alterations (He et al., 2020), however, the underlying mechanisms through which AS-IV alleviates constipation and its dependency on gut microbiota remains to be elucidated.

Abnormalities in the enteric nervous system (ENS) are a crucial mechanism underlying STC, with changes in neurotransmitters such as serotonin being one pathway. During peristaltic contraction, 90% of serotonin is released by enterochromaffin cells (ECs) (Keating and Spencer, 2019). Previous research indicates that Piezo2 protein, which is widely expressed in colonic ECs, is linked to serotonin secretion (Alcaino et al., 2018). In mice, age-related reductions in Piezo2-dependent tactile sensitivity can delay gastrointestinal transit (Treichel et al., 2022). Bidirectional communication between ECs and gut microbiota has been reported, with changes in microbiota potentially modulating ECs and impacting various gut physiological processes, including motility, absorption, and secretion (Wei et al., 2022). Building on these findings, our study investigates whether an association exists between gut microbiota alteration and ECs in the mechanism underlying AS-IV’s efficacy in alleviating STC.

In this study, building upon our previous work (He et al., 2020), we established comprehensive animal models of STC (normal microbiota, microbiota-depleted and fecal microbiota transplantation (FMT)) to explore the potential mechanism of AS-IV treatment in improving defecation. We observed the changes in mouse defecation and collected feces and intestinal tissues. We performed fecal 16S rDNA analysis and metabonomic sequencing in colon tissues and assessed changes in ECs and Piezo2 protein levels in ECs *in vitro*.

## Materials and methods

### Reagents

AS-IV (Cat. DH0015) was purchased from Chengdu Desite Bio-Technology Co., Ltd. (Chengdu, China) and dissolved in DMSO to prepare a 100 mM solution stored at −20°C. Similarly, 3-bromotyrosine (3-BrY) (Cat. S73497) and loperamide HCl (Cat. MB1460) were obtained from Yuanye Biotechnology Co., Ltd. (Shanghai, China) and Meilun Biotechnology (Dalian, China), respectively. They were separately dissolved in DMSO to obtain 100 mM and 40 mM stock solutions, respectively, and stored at −20°C. Antibodies used included ERK (Cat. #4695), phospho-ERK (Cat. #4370), p38 MAPK (Cat. #54470), and phospho-p38 MAPK (Cat. #4511) and GAPDH (Cat. #92310) from Cell Signaling Technology (United States); Piezo2 (Cat. NBP1-78624) from Novus Biologicals (Beijing, China); Caspase-3 (Cat. A5013), Caspase-3 p12 (Cat. A5357), Bcl-2 (Cat. A5010), and Bax (Cat. A5131) from Bimake (Houston, Texas, United States); and *β*-Actin (Cat. BS6007M) from Nanjing Bioworld Biotechnology Co., Ltd. (Nanjing, China). Goat anti-rabbit IgG H&L (HRP) (Cat. 511203) and goat anti-mouse IgG H&L (HRP) (Cat. 511103) were obtained from Zen BioScience (Chengdu, China).

### Animals and ethics statement

SPF-grade Kunming mice (n = 80, 40 males and 40 females), aged 4–6 weeks and weighing 20–25 g, were procured from Beijing Weitong Lihua Laboratory Animal Technology Co., Ltd. (Beijing, China, Certificate: SCXK (jing) 2021-0011) and housed at the Laboratory Animal Center of the Sixth Affiliated Hospital of Sun Yat-sen University (Guangdong, China). Mice were acclimated to the environment for 1 week with *ad libitum* access to food and water, under a controlled environment of 25°C ± 1°C, relative humidity of 50%–60%, and a 12-h light/dark cycle. All experimental procedures were approved by the Ethics Committee of the Laboratory Animal Center of the Sixth Affiliated Hospital of Sun Yat-sen University (Certificate: IACUC-2022012803) and were conducted in accordance with the Guidelines for the Care and Use of Laboratory Animals (National Institutes of Health, United States). Mice were randomly assigned to normal microbiota control (n = 30) or broad-spectrum antibiotic cocktail (0.2 q/L of ampicillin, neomycin and metronidazole and 0.1 q/L of vancomycin, ABX) (Zhao et al., 2020) treated group (n = 50) to depleted gut microbiota for subsequent experiments.

### Experimental design in SPF mice

Thirty mice (15 males and 15 females) were randomly assigned into three groups: a normal control (Nor) group, a constipation model (Lop) group that received loperamide (10 mg/kg bw/d), and an experimental (Lop + AS) group that received loperamide (10 mg/kg bw/d) and AS-IV (30 mg/kg bw/d) for 5 days, starting from the sixth day of loperamide administration. The Nor group received 0.5% sodium carboxymethyl cellulose (CMC) solution (0.5% CMC, 10 mL/kg) by oral gavage twice a day for 10 days, while Lop and Lop + AS groups were orally gavaged with a solution of loperamide (10 mg/kg bw/d) dispersed in 0.5% CMC for 10 days. The Lop + AS group received AS-IV solution (30 mg/kg bw/d) dispersed in 0.5% CMC by oral gavage 1 h after each loperamide administration. On the 11th day, stool, serum, cecal contents, and whole intestinal tissue were collected for analysis. Daily records of weight, food and water intake, and defecation weight were maintained, and fecal moisture content was determined on days 1, 5, and 10. Intestinal propelling rate of ink was measured on the 11th day using three mice from each group.

### Intestinal propelling rate test

Four mice per group were orally administered 25% Indian ink. After 20 min, the mice were euthanized, and their intestines were separated and measured for length. The distance between the pylorus and the front edge of the charcoal powder was recorded as the “ink pushing distance.” The intestinal propulsion rate was calculated as [toner travelling distance (cm)/total intestinal length (cm)] × 100%.

$$Intestinal\;propulsion\;rate=\frac{Toner\;travelling\;distance}{Total\;intestinal\;length}\times 100\%$$

### Experimental design in microbiota depleted mice

Thirty mice (15 males and 15 females) were randomly assigned to three groups of equal sex distribution: a microbiota-depleted normal control group (ABX), a microbiota-depleted constipation model group (ABX + Lop) receiving loperamide (10 mg/kg bw/d), and a microbiota-depleted experimental group (ABX + Lop + AS) receiving loperamide (10 mg/kg bw/d) and AS-IV (30 mg/kg bw/d). After a 1-week acclimation period, the mice were administered ABX water for 5 days. The ABX group received 0.5% CMC (10 mL/kg bw/d) by oral gavage twice a day for 10 days, while the ABX + Lop and ABX + Lop + AS groups received loperamide (10 mg/kg bw/d) dispersed in 0.5% CMC by oral gavage twice a day for 10 days. The ABX + Lop + AS group was cotreated with AS-IV during loperamide administration for 5 days, with the AS-IV (30 mg/kg bw/d) solution dispersed in 0.5% CMC and given by oral gavage 1 h after each loperamide administration. On the 11th day, stool and serum samples were collected, and data were analyzed as in the SPF mouse experiment.

### Experimental design in fecal microbiota transplantation mice

Twenty mice, consisting of ten males and ten females, were randomly assigned to the Lop-FMT + Lop + AS group and the AS-FMT + Lop + AS group, while the Lop-FMT group represents the FMT of microbiota obtained from Lop group mice into the ABX mouse model, while the AS-FMT group follows a similar procedure using microbiota derived from the Lop + AS group. After a 1-week acclimation period, the mice were administered ABX water for 5 days to eliminate gut microbiota. Both groups received loperamide (10 mg/kg bw/d) twice a day for 10 days, and starting from the sixth day, they received transplanted fecal bacteria once a day for 5 days, respectively. The transplanted fecal bacteria were prepared from daily collected fecal samples of the Lop and Lop + AS groups and used to make Lop-FMT and AS-FMT, which were given to the Lop-FMT + Lop + AS and AS-FMT + Lop + AS groups by gavage, respectively. Both groups were also treated with AS-IV (30 mg/kg bw/d) for 5 days, administered 1 hour after each gavage. On the 11th day, the mice were sacrificed, and stool and serum were collected for analysis.

### Tissue histological and immunohistological study

Ileum, colon, and cecum samples from mice were fixed in 4% paraformaldehyde for 12 h, embedded in paraffin, and subjected to histopathological examination. Hematoxylin and eosin (H&E) staining was performed to assess pathological changes. Image analysis was conducted using ImageJ 1.52i software on a Leica DM3000 LED research microscope. Immunohistochemical staining was performed to analyze the expression of c-Kit (CD117) and chromogranin A (CgA) in intestinal tissue, using an automated immunohistochemical stainer (Dako Autostainer Link 48). Tissue sections were incubated with c-Kit (Dako Denmark A/S, Ready-to-use type) (1:1) or CgA antibody (Dako Denmark A/S, Ready-to-use type) (1:1), followed by a secondary antibody (Dako Denmark A/S, Ready-to-use type) (1:1) and DAB horseradish peroxidase for color development. Hematoxylin counterstaining was performed before observation under a light microscope (Leica DM3000 LED, Wetzlar, Germany), and muscle thickness measurements of the ileum, colon, and cecum of mice were performed using ImageJ 1.52i software (Wayne Rasband National Institutes of Health, United States).

### Gut microbiota analysis

Genomic DNA was extracted from the samples using either CTAB or SDS methods, and subsequently assessed for purity and concentration through agarose gel electrophoresis. The DNA was then diluted to a concentration of 1 ng/μL with sterile water and amplified using specific primers with barcodes to target the sequencing region. The Phusion^®^ High-Fidelity PCR Master Mix, along with GC Buffer and a high-efficiency high-fidelity enzyme, were utilized to ensure efficient and accurate amplification. Bacterial diversity was identified using the 16S V4 region primers (515F and 806R), with PCR products analyzed *via* electrophoresis using a 2% agarose gel. Equal amounts of PCR products were combined, purified using the Qiagen Gel Extraction Kit, and then library construction was completed using the TruSeq^®^ DNA PCR-Free Sample Preparation Kit. The library was quantified *via* Qubit and Q-PCR, and sequencing was performed on the NovaSeq6000 platform once a qualified library was obtained.

### Microbiota metabolomics analysis

Prior to analysis, each sample (20 mg) was thawed on ice and mixed with a 400 μL solution of an internal standard (methanol: water = 7:3, V/V). The mixture was then vortexed for 3 min, sonicated in an ice bath for 10 min, and vortexed again for 1 min. After being placed at −20°C for 30 min, the mixture was centrifuged at 12,000 rpm for 10 min (4°C) to remove sediment. The supernatant was then further centrifuged at 12,000 rpm for 3 min (4°C), and a 200 μL aliquot was transferred for LC-MS analysis. The LC-ESI-MS/MS system included a UPLC, ExionLC AD, and QTRAP^®^ System. The sample extracts were separated on a Waters ACQUITY UPLC HSS T3 C18 column (1.8 µm, 2.1 mm*100 mm) with a gradient elution of water (0.1% formic acid): acetonitrile (0.1% formic acid) at a flow rate of 0.4 mL/min. The analytical conditions were optimized with a column temperature of 40°C, an injection volume of 2 μL, and a specific gradient program. The mass spectrometer was operated in positive and negative ion modes, with ion source parameters set at specific values. Instrument tuning and mass calibration were performed using QQQ and LIT modes, respectively. MRM transitions were monitored for each metabolite eluted during the analytical time period.

### Cell viability

QGP-1 cells, a human pancreatic endocrine cell line, serve as a model for human enterochromaffin cells, exhibiting high expression of transient receptor potential ankyrin 1 (TRPA1) and various ECs cell marker genes, including tryptophan hydroxylase 1 (TPH1), CgA, synaptophysin, *etc.* (Doihara et al., 2009b). The effect of loperamide and AS-IV on cell viability was assessed using the CCK-8 kit (APExBIO, K1018). QGP-1 cells were seeded at a density of 1 × 10^4^ cells per well in 96-well plates (5 × 10^3^ cells/well) and incubated for 24 h. The cells were then treated with AS-IV alone (0–100 μM) for 24 h, loperamide alone (0–50 μM) for 24 h, and a combination of loperamide (0–80 μM) with AS-IV (0–49 μM) for 24 h. After the treatment, 20 μL of CCK-8 solution was added to each well and incubated for 3 h. The absorbance was measured at 450 nm using an enzyme-labeled apparatus (Thermo Fisher Scientific, Waltham, MA, United States).

For protein analysis, cell lysis was performed using RIPA buffer (Meilunbio, MA0151) supplemented with protease and phosphatase inhibitors (Bimake, Houston, Texas, United States). Protein concentration was determined using a Bio-Rad assay kit (Bio-Rad Laboratories, Hercules, CA, United States). Electrophoresis was conducted on SDS polyacrylamide gels with varying concentrations (8%, 10%, and 15%), followed by transfer to PVDF membranes. The membranes were blocked with 5% skim milk powder for 1 h at room temperature and then incubated overnight at 4°C with primary antibody diluent. After washing with 0.1% TBST, protein bands were visualized using chemiluminescence and a luminescence imaging system (Bio-Rad Laboratories, Hercules, CA, United States). Secondary antibodies were used for 1 h at room temperature prior to visualization.

### Cellular immunofluorescence staining

QGP-1 cells (10^5^ cells/dish) were treated with loperamide (40 μm) in combination with either AS-IV (50 μm) or 3-BrY (50 μm) for 1 day in small confocal dishes. Following treatment, the cells were fixed in methanol for 10 min, permeabilized with 0.1% Triton X-100 for 20 min and blocked with 2% BSA for 20 min. Primary antibody was added overnight at 4°C, Piezo2 (1:200), TPH1 (1:200), followed by incubation with goat anti-rabbit IgG H&L (Alexa Fluor^®^ 555) (1:200) secondary antibody (Abcam, Shanghai, China, cat. ab150078) for 1 h at room temperature. After DAPI staining, the cells were imaged using a Leica laser confocal fluorescence microscope (Germany) and sealed with an anti-fluorescence quenching agent.

### Western blot analysis

Protein analysis was conducted using RIPA buffers (Meilunbio, MA0151) supplemented with protease and phosphatase inhibitors (Bimake, Houston, Texas, United States) for cell lysis. Protein concentrations were determined using a Bio-Rad assay kit (Bio-Rad Laboratories, Hercules, CA, United States). Electrophoresis was performed on SDS polyacrylamide gels of varying concentrations (8%, 10%, and 15%), followed by transfer to a PVDF membrane. The membrane was blocked with 5% skim milk powder at room temperature for 1 h and then incubated overnight at 4°C with primary antibodies. The primary antibodies used were as follows: ERK (Cat.#4695) (1:2000), phospho-ERK (p-ERK) (Cat.#4370) (1:2000), p38 MAPK (Cat.#54470) (1:2000), and phosphor-p38 (p-p38) MAPK (Cat.#4511) (1:2000) from Cell Signaling Technology (United States); Piezo2 (Cat.#NBP1-78624, Novus Bioicals, Beijing, China) (1:500); TPH1 (Cat. #BS3727, Bioworld, Nanjing, China) (1:500); Caspase-3 (Cat.#A5013) (1:2000), Caspase-3 p12 (Cat.#A5357) (1:2000), Bcl-2 (Cat.#A5010) (1:2000), and Bax (Cat.#A5131) (1:2000) from Bimake, Houston, Texas, United States. *β*-Actin (Cat.#BS6007m) (1:5000) was used as the internal reference gene. The secondary antibody was incubated at room temperature for 1 h at a dilution of 1:5000. Finally, enhanced chemiluminescence was used, and the membrane was imaged using a luminescent imaging system (Bio-Rad Laboratories, Hercules, CA, United States).

### Statistical analyses

The results, presented as mean ± standard deviation, were obtained from three independent experiments. Statistical analysis was performed using GraphPad Prism (San Diego, CA, United States) with one-way analysis of variance (ANOVA) and Tukey’s test. Significance was determined as *p* < 0.05 and denoted as *p < 0.05, **p < 0.01, ***p < 0.001, and ****p < 0.0001.

## Results

### AS-IV improves constipation in STC mice induced by loperamide

**FIGURE 1:**
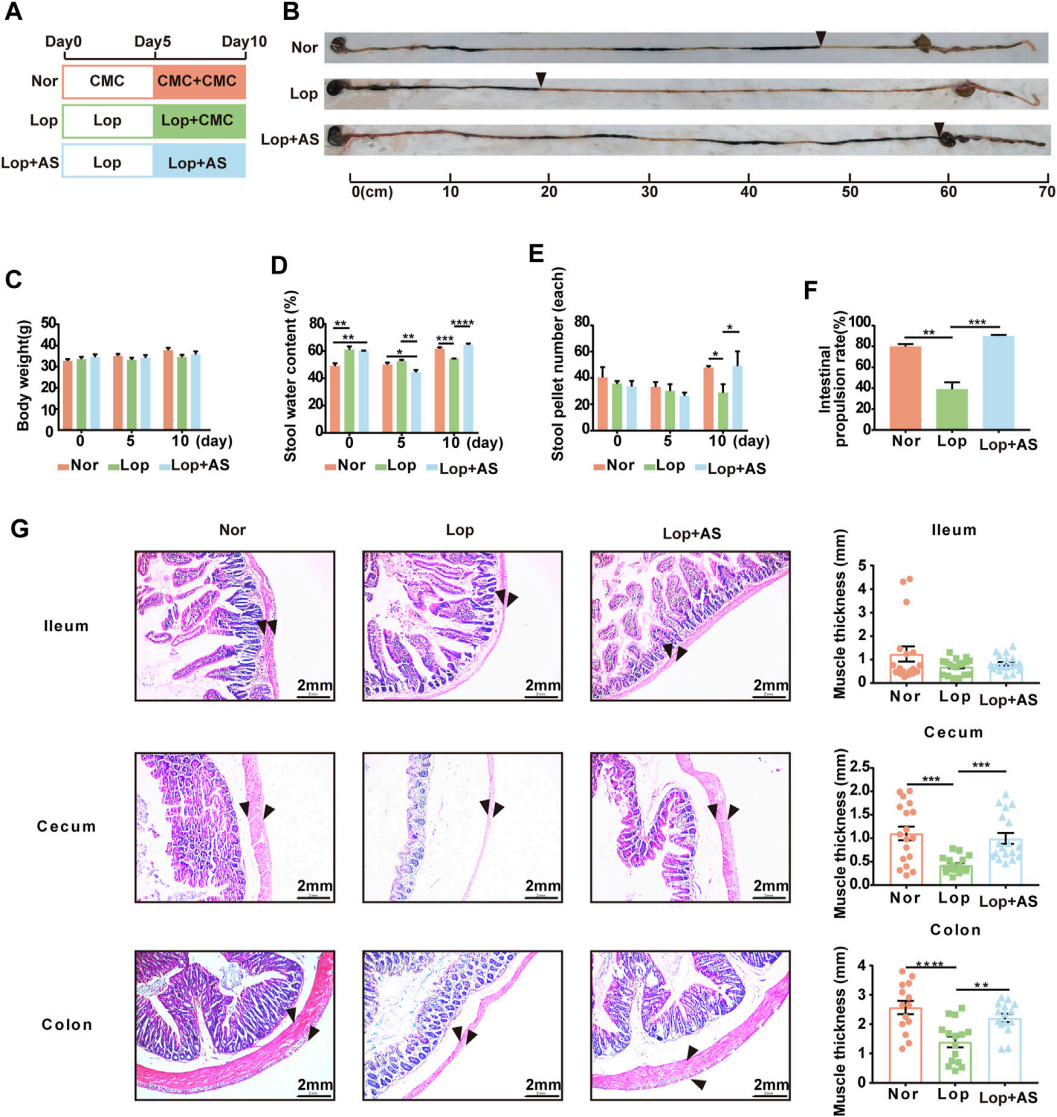
Establishment and characterization of the STC mouse model with astragaloside IV **(A)** Schematic diagram showing the establishment of the STC model. Thirty KM mice were randomly divided into three groups: Nor (normal control), Lop (loperamide 10 mg/kg bw/d), and Lop + AS (loperamide 10 mg/kg bw/d + astragaloside IV 30 mg/kg bw/d). **(B)** Results of the ink propulsion experiment conducted in the three groups of mice to assess gastrointestinal motility **(C)** Changes in body weight of KM mice on days 0, 5, and 10 of the experiment. **(D)** Fecal water content of KM mice during different experimental periods **(E)** Number of fecal particles in KM mice during different experimental periods. **(F)** Statistical graphs illustrating the intestinal propulsion rate of the ink propulsion experiment in the three groups. **(G)** Representative hematoxylin and eosin (H&E) staining results of intestinal tissue samples. Nor: normal control group; AS: astragaloside IV; Lop: loperamide; CMC: sodium carboxymethyl cellulose. Data are presented as mean ± SEM (*n* = 10). Significance was determined as *p* < 0.05 and denoted as *p < 0.05, **p < 0.01, ***p < 0.001, and ****p < 0.0001.

The mice were divided into three groups (Figure 1A), and AS-IV treatment significantly increased intestinal propulsion rate (Figure 1B). Body weight (Figure 1C) did not differ significantly between the groups throughout the experiment. At day 5 of STC modeling, the mice exhibited reduced water and food intake due to loperamide administration. However, by day 10, there were no significant differences in water intake among the groups, while the Lop group showed a notable decrease in food intake (Supplementary Figures S1A, B). By day 10 of treatment, the intestinal propulsion rate in the Lop groups was significantly reduced compared to the first day, indicating successful establishment of the STC model in mice. Additionally, on day 10, stool water content, stool pellet number, and intestinal propulsion rate were significantly decreased in the Lop group but significantly increased in the Lop + AS group compared to the Nor group (Figures 1D–F). These findings suggest that AS-IV effectively promotes fecal excretion and enhances gastrointestinal motility in Lop-induced constipation mice.

Based on Figure 1G, pathological evaluation via H&E staining demonstrated that the Lop group had more prominent intestinal lesions compared to the Nor group. These lesions were characterized by significant degeneration of the longitudinal serosa layer, decreased muscle thickness, increased intercellular space within the muscle layer, and disordered loose connective tissue in the submucosa. Additionally, the *U*-shaped concave space of the mucosal layer was found to be enlarged. The resulting data was statistically analyzed. The study found no significant difference between the Lop and Lop + AS groups in the ileal tissue, indicating that AS-IV’s therapeutic effect on the loperamide-induced constipation model is less reactive in ileal tissue. However, in colon and cecum tissue, muscle thickness was significantly reduced in the Lop group compared to the Nor group, whereas the Lop + AS group showed a significant increase in muscle thickness compared to the Lop group.

### Gut microbiota impact on AS-IV’s effects on intestinal peristalsis in mice

**FIGURE 2:**
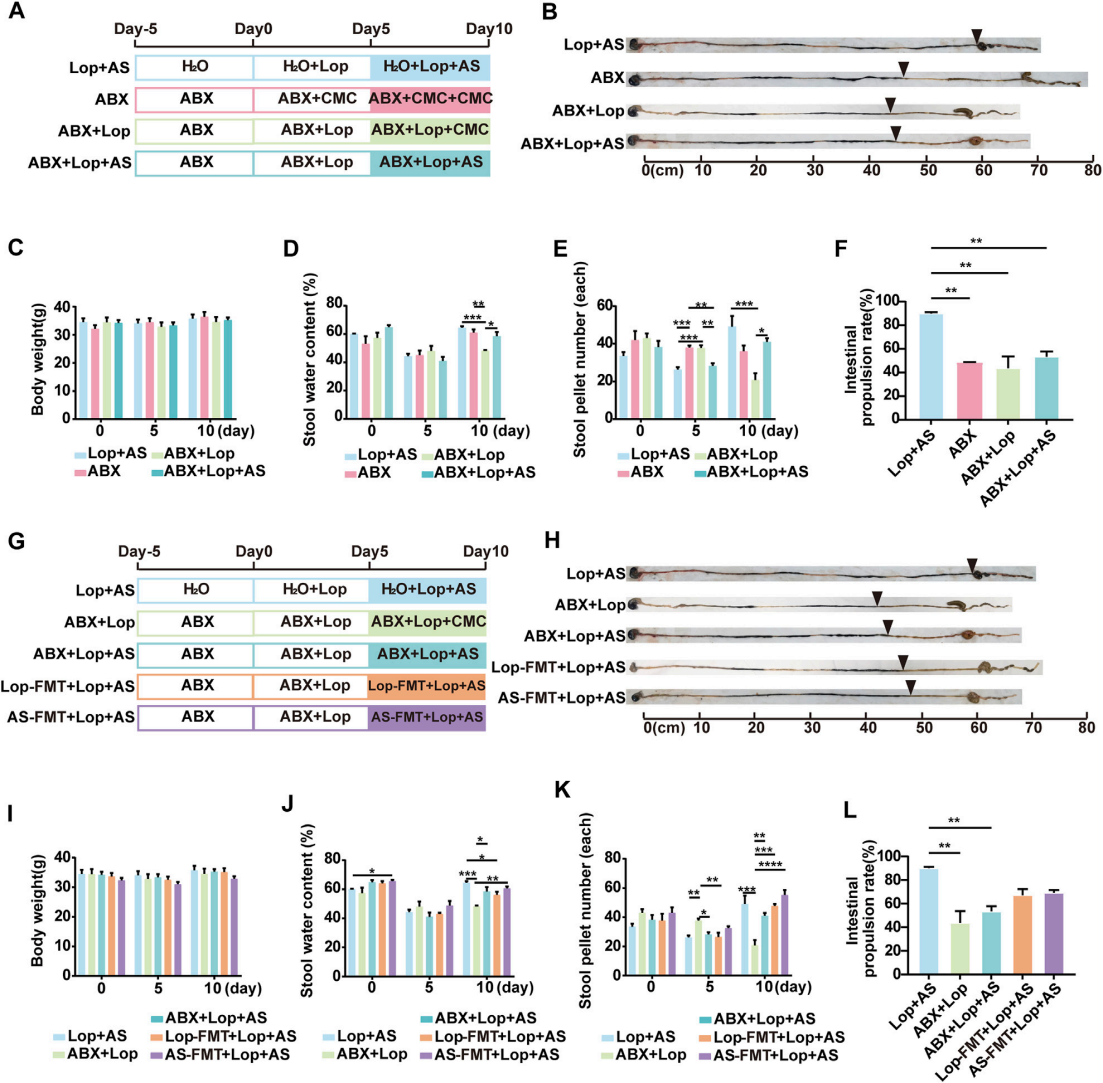
Establishment of a mouse model for studying enteric flora and the effects of astragaloside IV and loperamide on intestinal motility **(A)** Schematic diagram illustrating the establishment of a model for studying enteric flora in mice. Thirty mice were randomly assigned to ABX, ABX + Lop (10 mg/kg, bw/d), and ABX + Lop + AS (30 mg/kg, bw/d) groups. Analysis was performed jointly with the Lop + AS group. **(B)** Results of the ink propulsion test in mice without intestinal flora. **(C–F)** Changes in body weight, fecal water content, fecal particles, and intestinal propulsion rate in mice without intestinal flora on days 0, 5, and 10 of the experiment. **(G)** Schematic diagram of the fecal bacteria transplantation experiment. Twenty mice were randomly divided into Lop-FMT + Lop + AS group (fecal bacteria transplanted from the Lop group) and AS-FMT + Lop + AS group (fecal bacteria transplanted from the Lop + AS group). Analysis was performed jointly with the Lop + AS, ABX + Lop, and ABX + Lop + AS groups. **(H)** Results of the ink propulsion test in the five groups of mice. **(I–L)** Changes in body weight, fecal water content, fecal particles, and intestinal propulsion rate in the five groups of mice during the carbon ink propulsion test on days 0, 5, and 10 of the experiment. Nor: normal control group; AS: astragaloside IV; Lop: loperamide; CMC: sodium carboxymethyl cellulose, ABX: antibiotic water; Lop-FMT: fecal bacteria suspension from the Lop group; AS-FMT: fecal bacteria suspension from the Lop + AS group. Data are presented as mean ± SEM (n = 10). Significance was determined as *p* < 0.05 and denoted as *p < 0.05, **p < 0.01, ***p < 0.001, and ****p < 0.0001.

The results shown in Figure 2A compare the effects of microbiota depletion in three different groups of mice, with those of SPF mice in the Lop + AS group. All mice were fed ABX sterile water to eliminate their gut microbiota, both at the beginning of the experiment and 10 days after dosing. During the ABX-STC modeling on day 5, we observed no significant differences in the mice’s drinking water intake (Supplementary Figure S1C). However, loperamide intervention leads to reduced food intake, both in the normal (Lop + AS group) and depleted of gut microbiota (ABX + Lop group) on day 5 (Supplementary Figure S1D). On the 10th day of administration, there were no significant differences in water intake among the groups lacking intestinal flora, although the mice without intestinal flora exhibited certain characteristics. While the body weight of ABX + Lop + AS group mice remained constant, their stool water content and stool pellet number tended to lower than that in the Lop + AS group, and their intestinal propulsion rate were significantly slower than that in the Lop + AS group (Figures 2B–F). This suggests that the effectiveness of AS-IV treatment was diminished when gut microbiota were absent. However, since loperamide could still decrease the intestinal propulsion rate in ABX + Lop group compared to ABX group, AS-IV still promoted bowel movement in microbiota depleted mice, besides the intestinal propulsion rate in Lop + AS group was much faster than that in ABX + Lop + AS group. Therefore, it appears that AS-IV has multiple mechanisms for promoting bowel function, with a stronger effect observed under the condition of a normal gut microbiota.

To assess the impact of gut microbiota on the promotive effects of AS-IV, we conducted fecal microbiota transplants from groups Lop and AS into groups Lop-FMT + Lop + AS and AS-FMT + Lop + AS respectively, followed by recovery of gut microbiota in a sterile environment (Figure 2G). On the last day of administration, fecal bacteria were detected in group Lop + AS, while group ABX + AS had lost gut microbiota, and groups Lop-FMT + Lop + AS and AS-FMT + Lop + AS had fewer transplanted gut microbiota than group Lop + AS. There were no significant differences in weight, water intake, and food intake between the groups (Figure 2I, Supplementary Figures S1E, F). On the fifth day of administration, stool water content and stool pellet number decreased in all groups but recovered after AS-IV treatment (Figures 2J, K). In Figure 2F, under gut microbiota depleted, there are no significant differences in the comparison of intestinal propulsion rates among the groups (ABX vs. ABX + Lop vs. ABX + Lop + AS groups) on day 10. This indicates that loperamide does not inhibit gastrointestinal motility in the gut microbiota depleted. Even more, under gut microbiota depletion, AS-IV treatment fails to restore intestinal motility to the levels observed with a normal gut microbiota. Figures 2H, L showed on day 10 FMT led to a partial recovery of intestinal motility compared to the normal gut microbiota condition. Although the intestinal propulsion rates of FMT groups was lower than that observed with a normal gut microbiota (Lop + AS group), the difference was not statistically significant. Moreover, when compared to the normal condition (Lop + AS group), the intestinal propulsion rates was higher than that observed with gut microbiota deleption (ABX + Lop and ABX + Lop + AS groups), but there was also no statistically significant difference. These results suggest that AS-IV treatment can partially restore intestinal motility through a synergistic effect mediated by the recovery of gut microbiota. However, it is not solely dependent on the presence of gut microbiota.

These preliminary findings suggest that AS-IV has a better intestinal peristalsis-promoting effect on STC mice with naturally growing and abundant gut microbiota than on those with a disturbed microbiota environment, and that it may play a synergistic role with gut microbiota in treating loperamide-induced constipation in mice.

### AS-IV’s effect on *Candidatus arthromitus* abundance in STC mouse feces

To explore the potential influence of gut microbiota on the efficacy of AS-IV in treating STC, we conducted a 16S rDNA microbial spectrum analysis to examine the gut microbiota of mice in the Nor, Lop, and Lop + AS groups. Alpha diversity analysis was performed on the samples at a 97% consistency threshold, revealing 502, 513, and 524 OTUs in the Nor, Lop, and Lop + AS groups, respectively.

**FIGURE 3:**
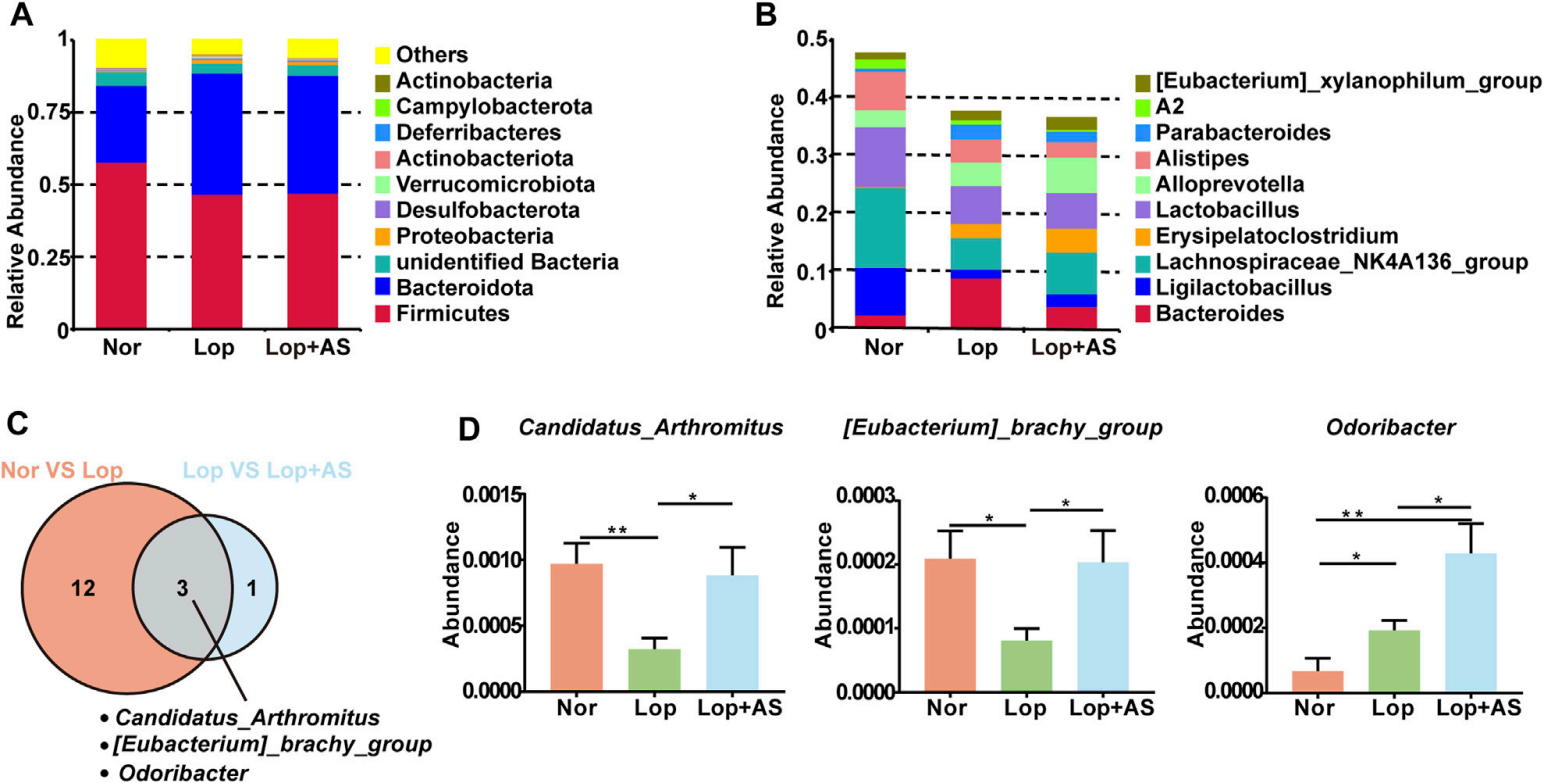
Species Abundance Analysis of Normal Control Group, Loperamide and Astragaloside IV Treated Groups **(A)** Histogram of relative abundance of species at the phylum level. **(B)** Histogram of relative abundance of species at the genus level **(C)** MetaStat analysis of species abundance data at the genus level. Venn diagram shows p ≤ 0.05 significant species **(D)** Statistical map of species significance between groups. Nor: normal control group; Lop: loperamide; AS: astragaloside IV. Significance was determined as *p* < 0.05 and denoted as *p < 0.05 and **p < 0.01.

Our study revealed changes in the relative abundance of disease-related gut microbiota. At the phylum level, *Firmicutes*, *Bacteroidota*, and *Unidentified Bacteria* were found to be dominant, with the top 10 species of highest abundance (Figure 3A). In Figure 3B, the dominant genera were *Bacteroides*, *Ligilactobacillus*, Lachnospiraceae *NK4A136, Lactobacillus, and Allopervotella*. Using MetaStat analysis at the genus level, we identified significant differences in species abundance between different groups and screened out *p* ≤ 0.05 for the Venn diagram (Figure 3C). *Candidatus arthromitus*, *Eubacterium brachy group*, and *Odoribacter* were identified as the significant different strains between Nor vs. Lop and Lop vs. Lop + AS. In Figure 3D, we present a list of microbiota genera that differed significantly between groups. Our findings indicate that *C. arthromitus* and Eubacterium brachy were reduced in loperamide-induced STC mice but restored by AS-IV treatment.

### AS-IV treatment alters the fecal metabolite profile in STC mice

**FIGURE 4:**
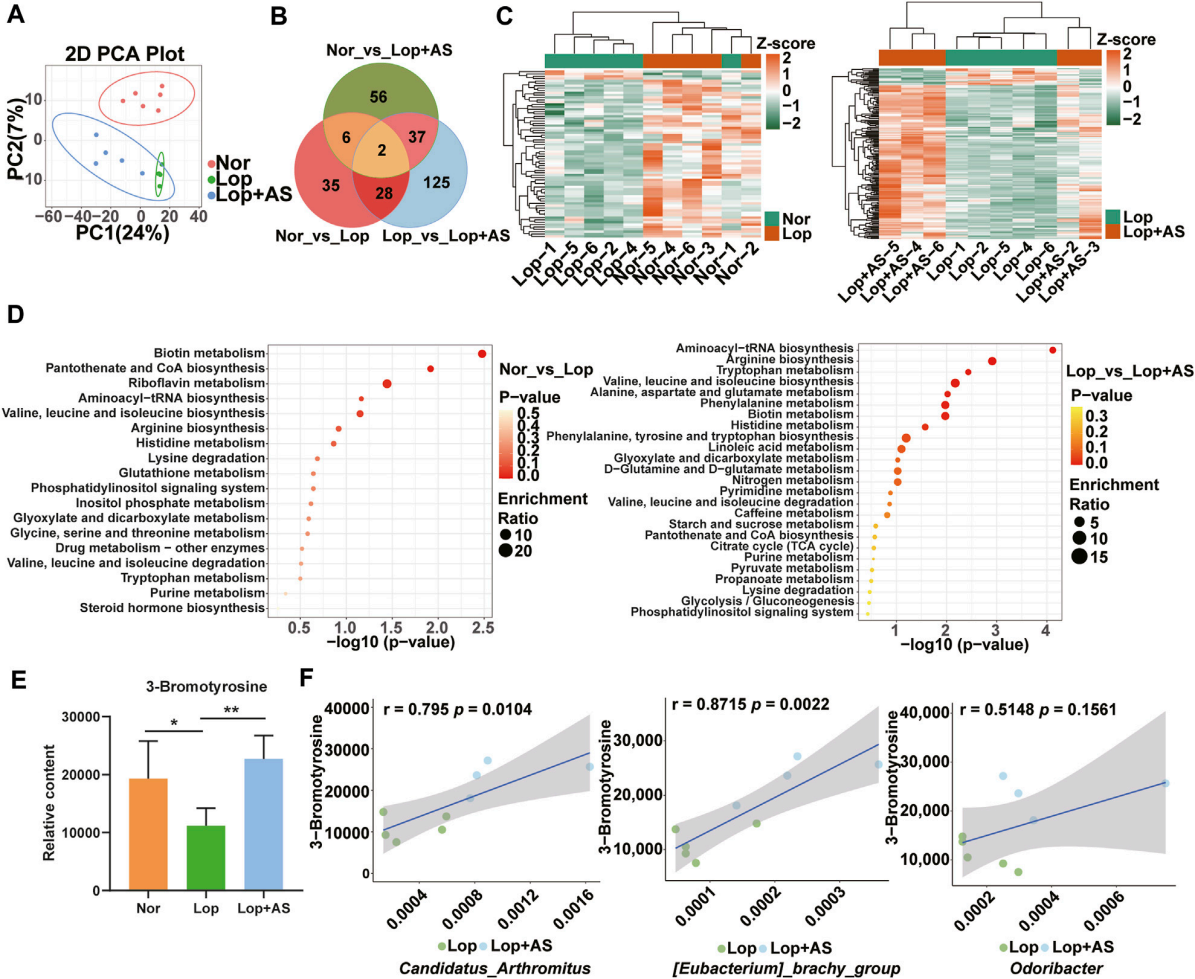
Metabolomic Analysis Reveals Differential Metabolites and Pathways in Loperamide-Induced Gut Dysbiosis and Astragaloside IV Intervention **(A)** Principal component analysis (PCA) of the metabolome. **(B)** Venn diagram showing differential metabolites **(C)** Heat maps illustrating clustering of metabolites in Nor and Lop groups; Heat maps illustrating clustering of differentiated metabolites in Lop and Lop + AS groups. **(D)** KEGG enrichment analysis of differentially expressed metabolites in Nor vs. Lop groups; KEGG enrichment analysis of differentially expressed metabolites in Lop vs. Lop + AS groups **(E)** Bar chart showing the relative content of 3-bromotyrosine **(F)** Scatter plot demonstrating the correlation between 3-bromotyrosine and differential microorganisms. Nor: normal control group; Lop: loperamide; AS: astragaloside IV. Significance was determined as *p* < 0.05 and denoted as **p < 0.01 and ***p < 0.001.

To investigate the underlying mechanisms by which AS-IV promotes bowel movement, we utilized UPLC-MS platform-wide target metabolome technology to detect metabolite changes in three groups of mice. In total, 1,232 first-class metabolites were identified, including amino acids and their metabolites, organic acids and their derivatives, fatty acyl, nucleotides and their metabolites, carbohydrates and their metabolites, glycerophospholipids, benzene and substituted derivatives, heterocyclic compounds, coenzymes, vitamins, bile acids, alcohols, amines, hormones, hormone-related compounds, tryptamines, cholines, pigments, sphingolipdis, and aldehydes, ketones, and esters. The PCA diagram (Figure 4A) indicates that Lop group tends to separate from the Nor and Lop + AS groups, respectively. There were 71 differential metabolites for Nor vs. Lop, 192 for Lop vs. Lop + AS, and 101 for Nor vs. Lop + AS (Figure 4B). Four downregulated and 67 upregulated metabolites were detected in groups Nor and Lop; 173 downregulated and 19 upregulated metabolites were detected in groups Lop and Lop + AS; 53 downregulated and 48 upregulated metabolites in groups Nor and Lop + AS. This finding suggests significant metabolic changes during the progression of the disease and its corresponding treatment.

The differential metabolites of Nor vs. Lop and Lop vs. Lop + AS were classified into two clusters based on inter-group differences, as shown in the clustering heat map (Figure 4C). KEGG enrichment map analysis revealed that differential metabolites in the biotin metabolism, pantothenate and CoA biosynthesis, and riboflavin metabolism pathways were mainly annotated and enriched in Nor vs. Lop groups (Figure 4D). The Aminoacyl-tRNA biosynthesis, arginine biosynthesis, and tryptophan metabolism pathways were the most enriched among differential metabolites in Lop vs. Lop + AS groups (Figure 4D).

We used the Wayne diagram screening method to select the differential metabolites between groups (Figure 4B). Subsequently, we selected differential metabolites with *p* < 0.05 that were common to both the Nor vs. Lop and Lop vs. Lop + AS comparison groups. Among them, we identified 3-bromothyronine (3-BrY), which is classified as a derivative of tyrosine (Figure 4E). Spearman rank correlation analysis revealed a significant correlation between 3-BrY and *C. arthromitus* (r = 0.795, *p* = 0.0104), *E. brachy group* (r = 0.8715, *p* = 0.0022), and *Odoribacter* (r = 0.5148, *p* = 0.1561) (Figure 4F).

### AS-IV protects against loperamide-induced ECs and interstitial cells of cajal loss in mouse colon

**FIGURE 5:**
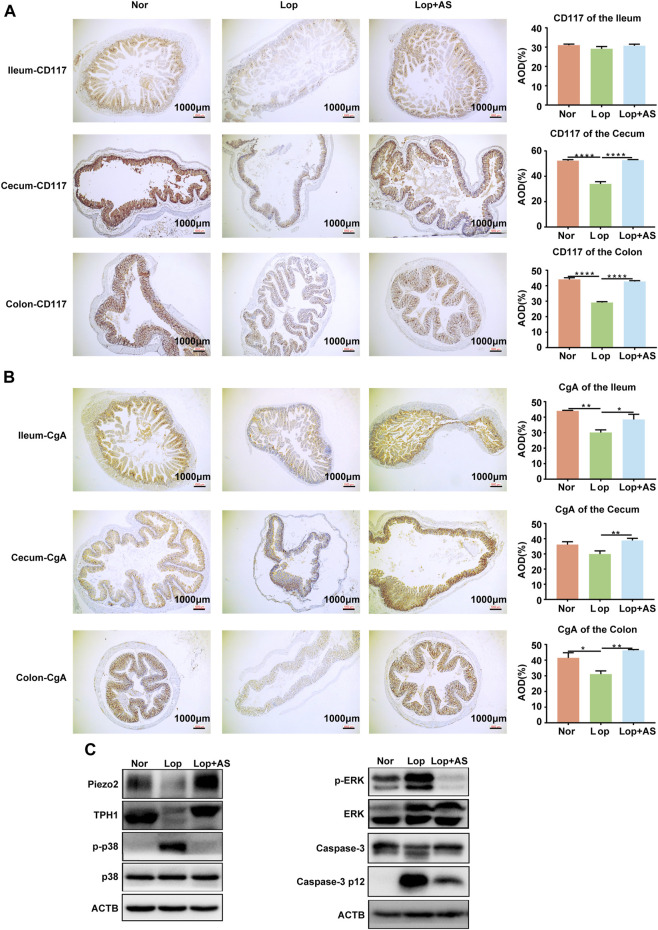
Immunohistochemical Analysis of CD117 and CGA Expression in the Colon and Western blot Analysis from Piezo2, TPH1, p-p38, p38, p-RRK, ERK, Caspase-3 p 12 and ACTB **(A, B)** Immunohistochemical staining of CD117 **(A)** and CGA **(B)** in the ileum, cecum, and colon tissues with corresponding average optical density (AOD) of the muscle layer in each tissue. **(C)** Western blot analysis of colon tissue from normal control group (Nor), loperamide-treated group (Lop), and astragaloside IV-treated group (AS). Nor: normal control group; Lop: loperamide; AS: astragaloside IV; ACTB: beta actin. Significance was determined as *p* < 0.05 and denoted as *p < 0.05, **p < 0.01, ***p < 0.001, and ****p < 0.0001.

The colon and cecum of STC mice induced by loperamide were evaluated for the impact of AS-IV in Figures 5A, B. Histological analysis revealed that AS-IV treatment predominantly affected the colon and cecum. CD117 is the specific cytochemical marker of Interstitial cells of Cajal (ICC) (Miettinen and Lasota, 2005). CgA protein was found to be colocalized with serotonin in ECs storage granules (Doihara et al., 2009b). Average optical density (AOD) values of CD117 and CgA were lower in the Lop group compared to the Nor group in both colon and cecal tissues. Treatment of AS-IV resulted in a significant increase in the expression of CD117 and CgA proteins in the colon and cecal tissues compared to Lop group (Figures 5A, B). The result suggests that AS-IV could restore colon ICC and ECs induced by loperamide in mice (Figure 5B).

The expression of TPH1, an ECs marker, was significantly decreased in the colon tissue of the Lop group compared to the Nor group. In the Lop + AS group, the expression of TPH1 was restored, and the expression of Piezo2 also exhibited corresponding alterations (Figure 5C, Supplementary Figure S2A). Western blot analysis revealed that loperamide increased p-p38 and p-ERK levels in mouse colon tissue, while AS-IV treatment reversed these effects (Figure 5C, Supplementary Figure S2A). These findings suggest that the mechanism underlying the promotion of bowel movement by AS-IV is associated with the p38 MAPK and ERK pathways. Furthermore, we conducted validation of caspase-3 in the colonic tissues of mice. The results revealed that loperamide inhibited the expression of caspase-3 while promoting the expression of caspase-3 p12 subunit, indicating the activation of the caspase-3-mediated apoptotic pathway by loperamide. Importantly, we observed a significant decrease in the expression of the caspase-3 p12 subunit in the colonic tissues after treatment with AS-IV, suggesting the inhibitory effect of AS-IV on loperamide-induced caspase-dependent cellular apoptosis in the colon tissue (Figure 5C, Supplementary Figure S2A).

### Regulation of MAPK signaling pathways by 3-Bry protects the survival of human ECs

**FIGURE 6:**
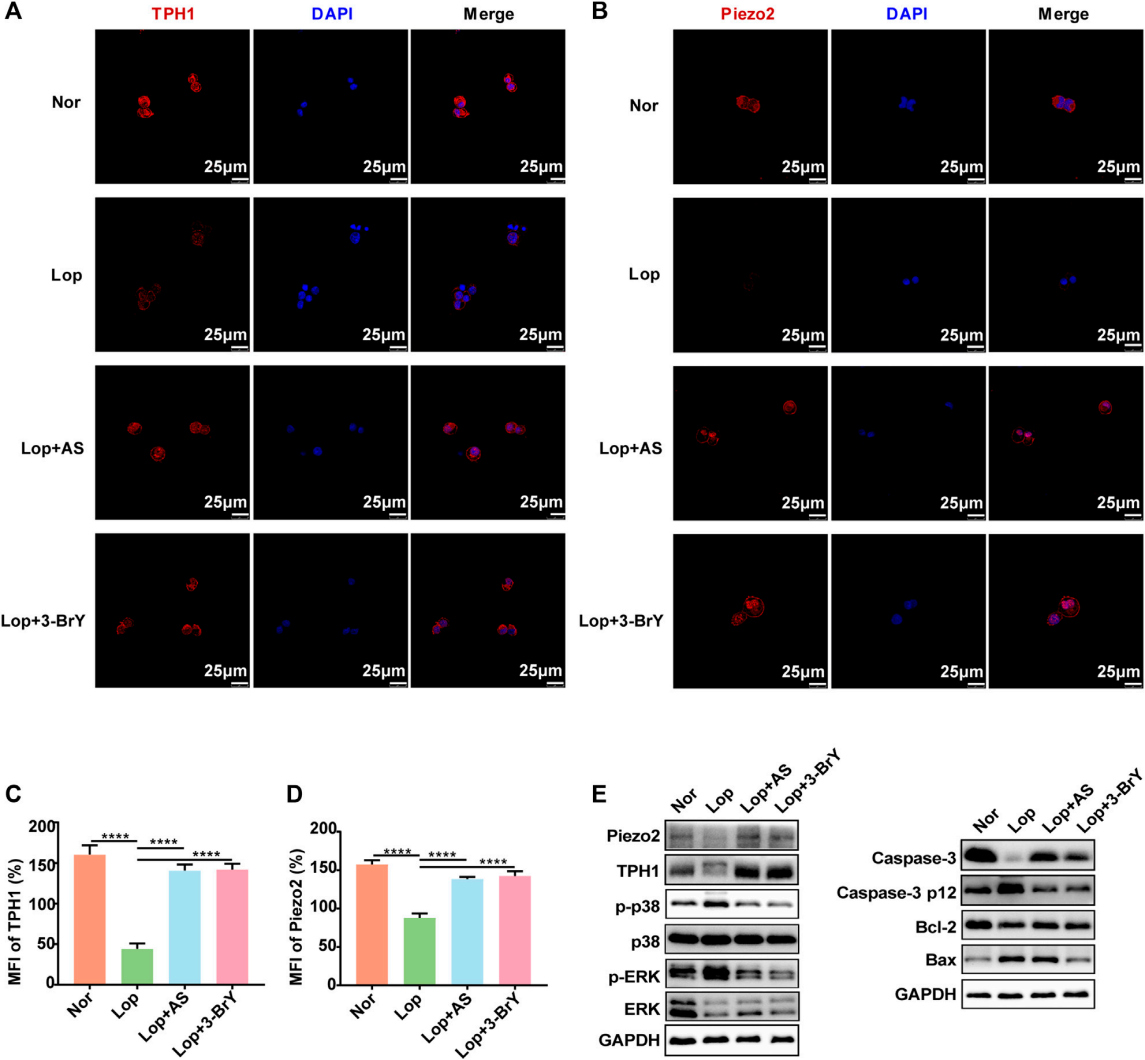
Effects of Loperamide, Astragaloside IV, and 3-bromotyrosine on TPH1 and Piezo2 expression in QGP-1 cells **(A, B)** Immunofluorescence staining was performed to evaluate the expression of TPH1 and Piezo2 in QGP-1 cells treated with Loperamide (40 μM), Astragaloside IV (50 μM), or 3-bromotyrosine (50 μM) for 24 h. TPH1 and Piezo2 were stained in red, while the nucleus was stained in blue. **(C, D)** Mean fluorescence intensity (MFI) of TPH1 and Piezo2 was quantified using ImageJ software. **(E)** Western blotting analysis was performed to evaluate the expression of QGP-1 cell proteins under different treatment conditions with loperamide (40 μM), Astragaloside IV (50 μM), or 3-bromotyrosine (50 μM) for 24 h. Nor: normal control group; Lop: loperamide; AS: astragaloside IV, 3-BrY: 3-bromotyrosine. Significance was determined as *p* < 0.05 and denoted as *p < 0.05, **p < 0.01, ***p < 0.001, and ****p < 0.0001.

To investigate the molecular mechanisms underlying the promotion of intestinal peristalsis by AS-IV and fecal metabolite 3-BrY, we cultured QGP-1 cells *in vitro*. We induced STC by treating the cells with loperamide for 24 h followed by treatment with AS-IV and 3-BrY to investigate changes in cell phenotype and relative protein levels. Immunofluorescence staining revealed that TPH1 expression was significantly reduced in ECs following loperamide modeling, which was reversed by AS-IV and 3-BrY treatment (Figures 6A, C). The expression of Piezo2, along with TPH1, suggests that AS-IV mitigates ECs damage induced by loperamide and increases Piezo2 expression (Figures 6B, D). A consistent trend was also observed in Western blot analysis (Figure 6E). Our CCK8 experiments demonstrated that AS-IV had no toxicity within 100 μM and increasing loperamide concentrations significantly reduced the number of ECs, with an IC50 of approximately 34.41 μM (Supplementary Figures S3A, B). However, the addition of AS-IV reduced the loperamide-induced cytotoxicity and promoted ECs proliferation (Supplementary Figures S3C, D). Loperamide resulted in a decrease in the expression levels of Bcl-2 and caspase-3, while it led to an increase in the expression levels of p-p38, p-ERK, Caspase-3 p12 subunit, and Bax. Conversely, both AS-IV and metabolite 3-BrY treatment exhibited inhibitory effects on the activation of the p38 MAPK and ERK signaling pathways induced by loperamide. And both AS-IV and metabolite 3-BrY also increased Caspase-3 expression and decreased Caspase-3 p12 subunit expression. In addition, AS-IV slightly decreased Bax and increased Bcl-2 expression. And 3-BrY significantly decreased the expression of Bax and relatively increased Bcl-2 (Figures 6E, Supplementary Figure S2B).

## Discussion

In this study, we discovered that AS-IV treatment improved intestinal mobility and reduced ECs loss in STC mice. Additionally, *in vitro* experiments showed that 3-BrY, a fecal metabolite linked to specific gut microbes, protected ECs from caspase-dependent apoptosis by inhibiting the p38 MAPK and ERK signaling pathways. Notably, our study employed a comprehensive approach, utilizing different mouse models (normal microbiota, microbiota-depleted, and FMT), to provide a more thorough understanding of AS-IV treatment, rather than relying solely on normal microbiota mouse models alone (Yao et al., 2022). Previous studies investigating the mechanism of AS-IV treatment for STC have primarily focused on the interplay between the gut microbiota, fecal metabolites, and research targeting ICC cells, while overlooking dedicated experimental investigations on ECs. It is worth noting that ECs play a pivotal role in regulating intestinal motility by secreting over 90% of human serum serotonin to modulate the enteric nervous system (ENS) (Bellono et al., 2017). Therefore, to explore the potential mechanisms of AS-IV in STC treatment and its interaction with ECs, we conducted initial *in vitro* validation using QGP-1 cells. In summary, our study aims to investigate the specific connection between the regulation of microbiota, fecal metabolites, and ECs during AS-IV treatment for STC.

By comparing the intestinal propulsion rates among different groups, we observed a marked suppression of intestinal motility in the STC model. However, AS-IV treatment led to increased fecal water content and total fecal output, resulting in the restoration of intestinal motility. These findings confirm the successful establishment of the STC model and the effectiveness of AS-IV in treating STC, consistent with previous research (He et al., 2020). Under microbiota-depleted condition, AS-IV still exhibited the ability to promote intestinal motility. However, in mice with naturally growing gut microbiota, AS-IV demonstrated a more pronounced effect on enhancing intestinal motility. Interestingly, in a microbiota-depleted environment, transplantation of gut microbiota further enhanced the effects of AS-IV, although it did not reach the level observed in mice with natural gut microbiota. Based on these findings, we can conclude that modulation of the gut microbiota is an important mechanism through which AS-IV promotes intestinal motility. However, it is likely that other mechanisms are also involved in this process.

Additionally, we observed significant differences in species abundance among different groups. In loperamide-induced STC mice, there was a decrease in *C. arthromitus* and *E. brachy group*, which was restored after AS-IV treatment. The enteric nervous system (ENS) structurally resembles the central nervous system and contains neurons that independently regulate bowel movements and brain-gut communication (Liu et al., 2009). ECs play a crucial role in producing and transmitting 5-HT, vital for enteric neuron development, maturation, and intestinal hormone regulation (Cook and Mansuy-Aubert, 2022). In mice, *C. arthromitus* is crucial for the postnatal maturation of innate and adaptive immune functions in the gut, inducing strong IgA responses (Klaasen et al., 1993) and recruitment and activation of intraepithelial CD8 T lymphocytes (Umesaki et al., 1995) and lamina propria CD4 T cells. Moreover, due to the close proximity of the innervated gastrointestinal tract and immune cells (Jakob et al., 2020), it is hypothesized that *C. arthromitus* may affect ECs function through immune modulation, which requires further exploration. Furthermore, the abundance of *C. arthromitus* is associated with compounds involved in tryptophan and bile acid metabolism. Overall, the results of this study suggest that changes in the microbial composition might have an impact on the intestinal immune status, potentially leading to improvements in gut permeability and promoting gastrointestinal motility.

Using UPLC-MS-based targeted metabolomics, we identified 3-BrY as a differential metabolite, and its abundance changes were strongly correlated with *C. arthromitus* and *E. brachy group*. *In vitro* cultivation of ECs (QGP-1) and establishment of an STC model using loperamide resulted in a significant decrease in the expression of TPH1 and Piezo2 in the cells. This indicates the potential of loperamide to inhibit ECs growth and reduce serotonin secretion. Currently, there is no established cellular model for constipation in enterochromaffin cells (ECs). Therefore, QGP-1 cells can be considered as a potential candidate model for loperamide induced in constipation research. On the other hand, treatment with AS-IV and 3-BrY increased the expression of TPH1 and Piezo2 in ECs, improving their viability, reducing loperamide-induced caspase dependent apoptosis, and promoting proliferation.

Piezo proteins are known to transduce mechanical signals and affect mitogen-activated protein kinase (MAPK) signaling processes, including the JNK, p38, and ERK pathways in dental cells (Gao et al., 2017). p38 kinase can inhibit cell proliferation by impeding cell cycle progression and can also act as an upstream mediator of apoptosis to induce apoptosis (Sui et al., 2014). Several studies have demonstrated that MAPK pathways can modulate Piezo2 currents (Doihara et al., 2009b). In addition, literature suggests that AS-IV has anti-inflammatory properties by inhibiting the phosphorylation of ERK1/2 and p38 in various diseases, such as atherosclerosis and hepatic steatosis (Gao et al., 2017; Del Rosario et al., 2020; Chen et al., 2021). Therefore, we investigated the activation of MAPK pathways in mouse colon and observed a significant increase in p-p38 and p-ERK levels in STC tissue, while their expression decreased in the Lop + AS group. These findings suggest that the mechanism underlying the promotion of bowel movement by AS-IV is associated with the p38 MAPK and ERK pathways. Western blot analysis of tissue showed that p38 and ERK pathways were activated upon AS-IV induced ECs, indicating that the protective effect of AS-IV was regulated by these two pathways. These results further support the notion that Loperamide reduces the number of ECs in the intestinal tissue of the STC mice, and AS-IV, on the other hand, enhances their survival.

Our experimental results indicate that AS-IV exerts a regulatory effect on the intestinal microecology, leading to enhanced colonization by *C. arthromitus*. Moreover, our research has revealed that 3-BrY, as a microbial metabolite during AS-IV treatment in loperamide-induced constipation mice, was a suppressor of caspase-dependent cellular apoptosis, providing cellular protection, and inhibiting the activation of the p38 and ERK signaling pathways. Additionally, 3-BrY upregulates the expression of Piezo2 protein, facilitating serotonin release, increasing mucosal secretion, and promoting intestinal peristalsis.

However, this study has some limitations. It only provides a preliminary discussion on the mechanism by which AS-IV promotes intestinal peristalsis in STC mice. The effects of AS-IV metabolite, 3-BrY, on ECs or other colon tissues and on the human body are not yet clear. Moreover, our study focused only on the closely related contents for further research, while 16S rDNA and metabonomic sequencing results revealed changes in various bacterial groups and metabolites, the interactions between these groups and their impact on the changes in intestinal tissue structure and function remain unclear, and requires further experimental discussion.

## Conclusion

This study suggests that AS-IV has potential therapeutic benefits in regulating intestinal microecology and promoting intestinal peristalsis. However, further research is necessary to elucidate the complete mechanism of action and its implications on human health.

## Data Availability

The datasets presented in this study can be found in online repositories. The names of the repository/repositories and accession number(s) can be found below: https://www.ncbi.nlm.nih.gov/, PRJNA949425.
