# Supplementary material for: Astragaloside IV improves slow transit constipation by regulating gut microbiota and enterochromaffin cells
Source: Front Pharmacol. 2023 Nov 21;14:1196210. doi: 10.3389/fphar.2023.1196210 (PMC10703044; doi:10.3389/fphar.2023.1196210)
Supplement: Supplementary file 1 [file DataSheet1.docx]

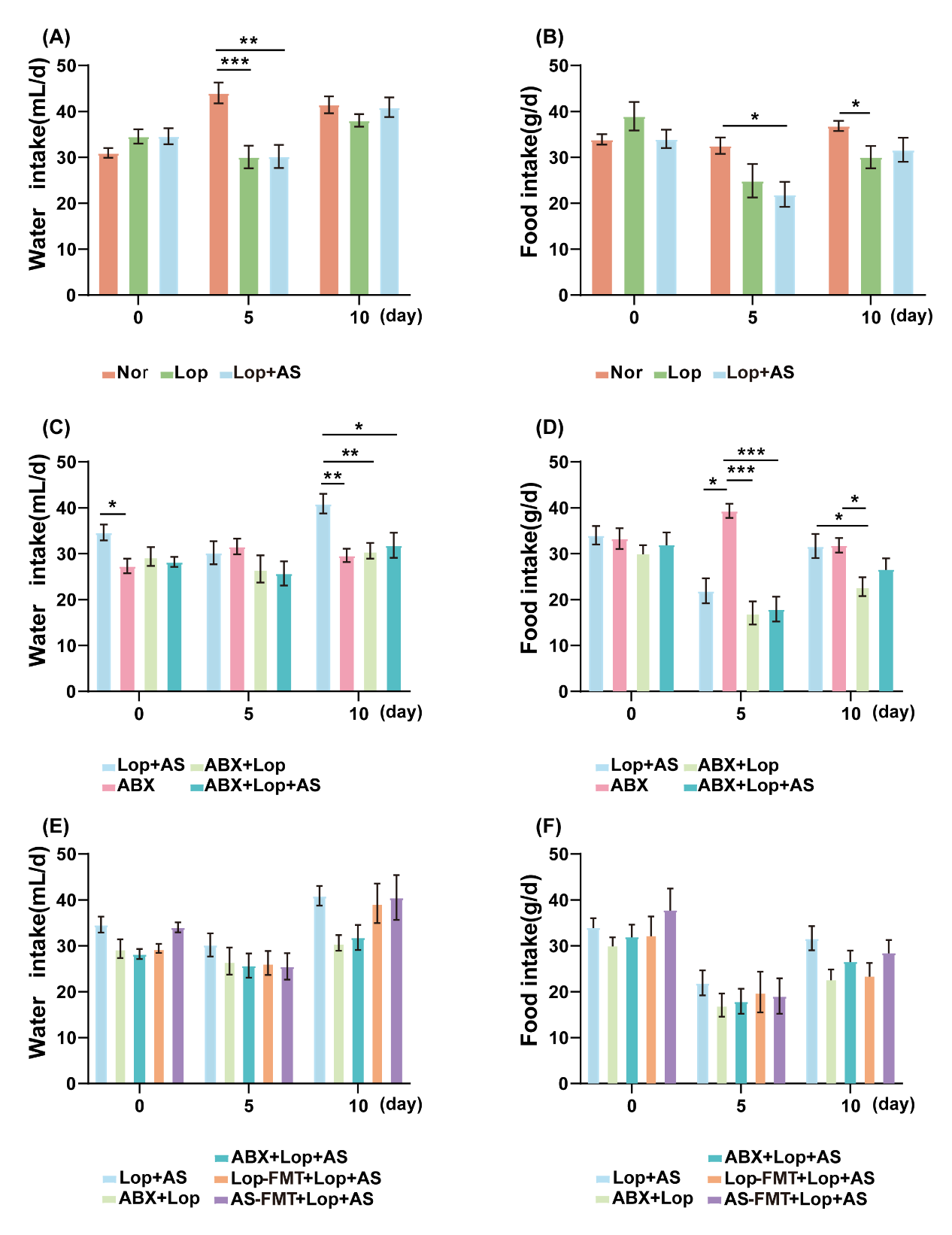
 **FIGURE S1 The histogram of water consumption and food intake of each group of mice on days 0, 5 and 10.**

Nor: normal control group; AS: astragaloside IV; Lop: loperamide; ABX: antibiotic water; Lop-FMT: fecal bacteria suspension from the Lop group; AS-FMT: fecal bacteria suspension from the Lop+AS group.

Significance was determined as *p* < 0.05 and denoted as **p* < 0.05, ***p* < 0.01, ****p* < 0.001, and *****p* < 0.0001.

**
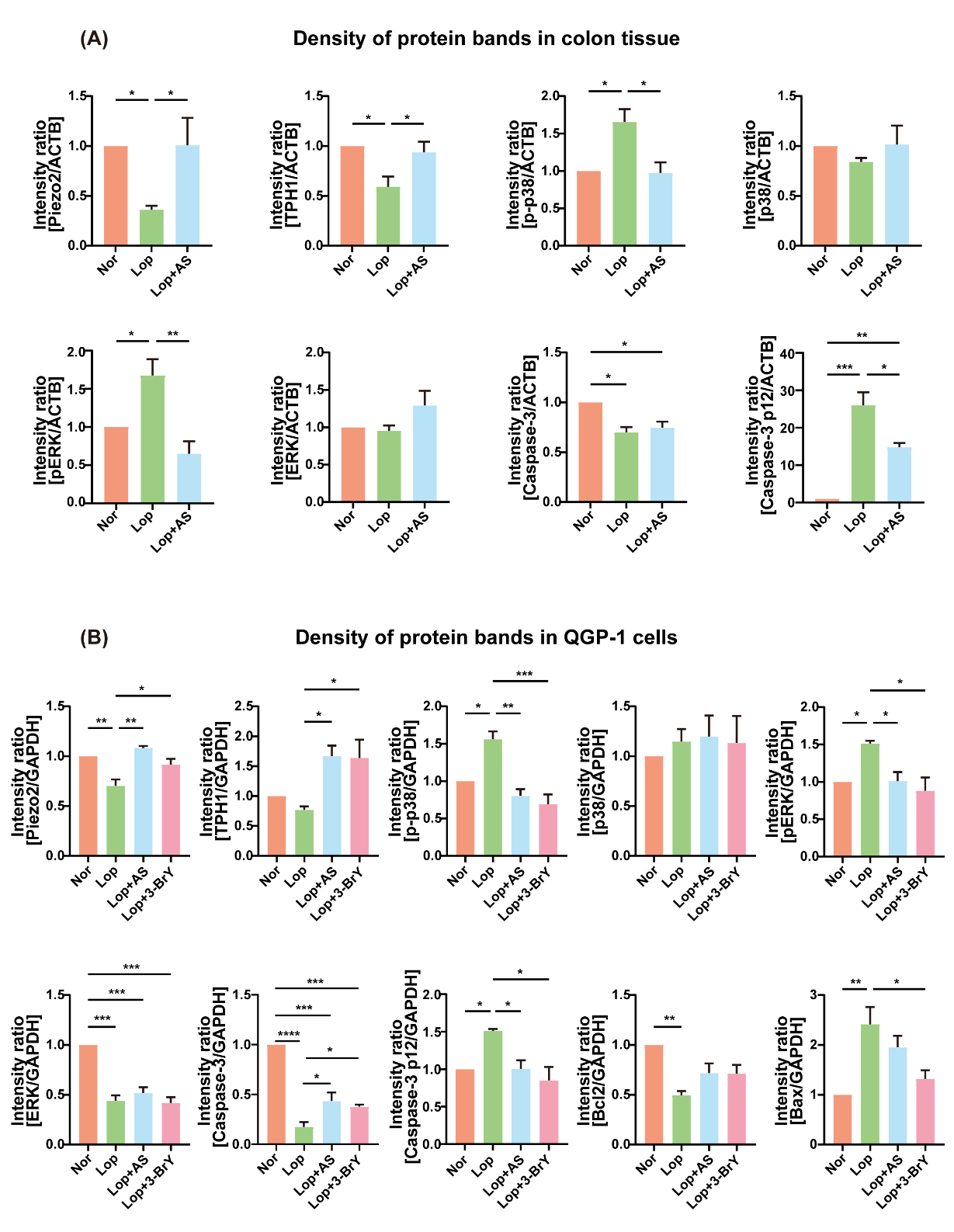
FIGURE S2 Density analysis of protein bands in colon tissue and QGP-1 cells by western blot**

**(A)** The intensity ratio of Piezo2, TPH1, p-p38, p38, pERK, ERK, Caspase-3, and Caspase-3 p12 bands in colon tissue, normalized to ACTB as the reference, as determined by western blot analysis.

**(B)**The intensity ratio of Piezo2, TPH1, p-p38, p38, pERK, ERK, Caspase-3, Caspase-3 p12, Bcl-2 and Bax bands in QGP-1 cells, normalized to GAPDH as the reference, as determined by western blot analysis.

Significance was determined as *p* < 0.05 and denoted as **p* < 0.05, ***p* < 0.01, ****p* < 0.001, and *****p* < 0.0001.


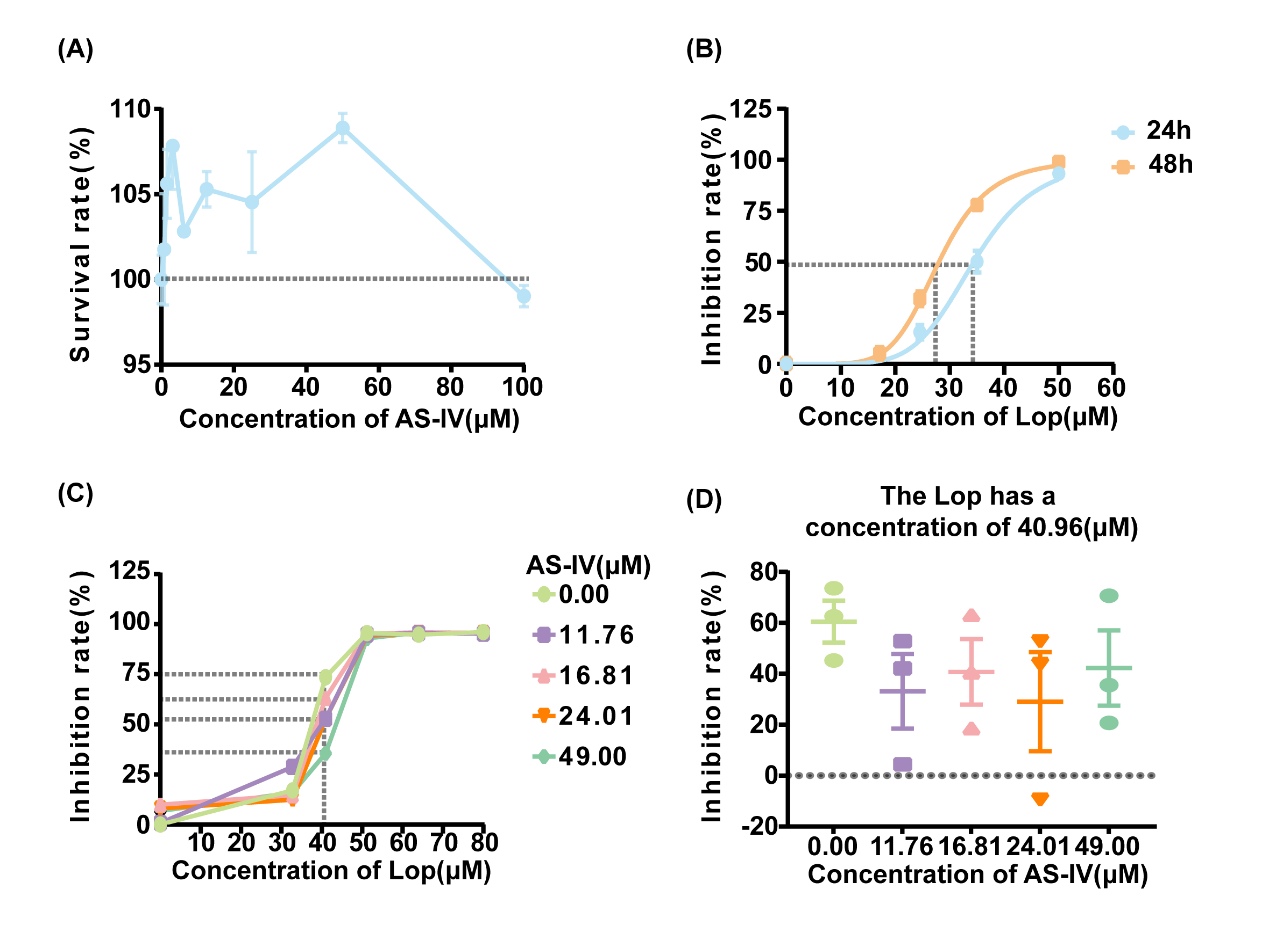


**Figure S3 Exploring the administration concentrations of Astragaloside IV and loperamide in QGP-1 cell line.**

(A) The cell survival rate was measured by CCK8 assay at different concentrations of Astragaloside IV (0-100μM).

(B) Cell lethality was measured by CCK8 assay at different concentrations of loperamide (0-50μM) for 24h and 48h, with IC50 values of 34.41 μM and 28.03 μM, respectively.

(C) Cell mortality was measured by CCK8 assay at different concentrations of loperamide (0-80μM) in the presence of Astragaloside IV (0-49μM).

(D) Cell fatality rate was measured by CCK8 assay at different concentrations of Astragaloside IV (0-49μM) when loperamide concentration was 40.96 μM.
